# Estimate of CRP and TNF-alpha level before and after periodontal therapy in cardiovascular disease patients

**DOI:** 10.11604/pamj.2013.15.92.2326

**Published:** 2013-07-10

**Authors:** Pradeep Koppolu, Satyanarayana Durvasula, Rajababu Palaparthy, Mukhesh Rao, Vidya Sagar, Sunil Kumar Reddy, Swapna Lingam

**Affiliations:** 1 Department of Periodontics, Sri Sai college of dental surgery, Vikarabad, AP, India; 2 Department of Periodontics, Kamineni institute of dental sciences, Narketpally, AP, India; 3 Yashoda hospital, Hyderabad, India; 4 Department of Oral medicine & Radiology, Sri Sai college of Dental Surgery,Vikarabad, AP, India

**Keywords:** CVD, CRP, TNF-alpha, lipopolysaccharide, periodontitis

## Abstract

**Introduction:**

Epidemiological studies show that individuals with periodontitis have a radically amplified threat to develop cardiovascular disease. CRP& TNF-α, are acute phase proteins monitored as a marker of inflammatory status, which have been identified as a major risk factor for atherosclerotic complications. Elevated CRP & TNF-α level in periodontitis patients have been reported by several groups. The present study was performed to determine whether presence of periodontitis and periodontal therapy could influence the serum levels of CRP & TNF-α in cardiovascular disease patients.

**Methods:**

Forty cardiovascular disease subjects participated in the study. They were classified into two groups. Group A (Control) where no periodontal treatment was given, Group B (Test) where periodontal treatment (scaling & root planing) was performed. Periodontal clinical parameters like OHI-S, probing pocket depth, were evaluated together with serum CRP, TNF-α, at baseline and reassessed after 8 weeks for all the subjects in both the groups.

**Results:**

The CRP & TNF-α levels in both the groups decreased but the decrease in the Group A was minimal and was not statistically significant (P > 0.05); whereas in Group B where periodontal therapy was performed, there was statistically significant decrease.

**Conclusion:**

It can be concluded from the study that there can be a possible causal relationship between pathogenesis of periodontal disease and CVD as inferred from the statistical significant outcome in the form of decreased inflammatory biomarkers after the periodontal treatment.

## Introduction

Periodontitis is an infectious disease coupled with a number of gram negative microorganisms, along with pathogenic bacteria, a susceptible host is also imperative. Substantiate evidence budding in the last decade has shed light on the contrary side of the relationship linking systemic health and oral health. Surmise worldwide trends in the burden of disease, principally cardiovascular disease (CVD), is aided by examining regional trends. Approximately 80% of the world′s population lives in the low and middle income nations, global rates of CVD are largely driven by rates of progression in these countries.

Quite a few reports have implicated long standing periodontal disease in the advancement of CVD, cerebrovascular accident and preterm low birth weight infants [1]. Periodontitis has been proposed as having a modulating role in CVD and adverse pregnancy outcome [2]. There is mounting evidence that inflammatory mechanisms as well as unremitting infections play a foremost part in atherogenesis and CVD. Quite a few studies propose a relationship linking periodontal diseases and atherosclerosis [3].

Numerous studies specify that the periodontal inflammation is not sternly a localized process, but may lead to systemic reorganization in the immune function. The localized inflammatory retort to periodontally pathogenic bacteria or bacterial products is characterized by permeation of the periodontal tissues by inflammatory cells. The aftermath of inflammatory progression of periodontitis systemically leads to production of mediators in the vicinity such as C-reactive protein (CRP), interleukins-1beta (IL-1β), tumor necrosis factor-alpha (TNF-α) and interleukin 6 (IL-6) i.e Proinflammatory cytokines [2].

Atherosclerosis is a progressive disease process in which large to medium sized arteries become occluded with fibrolipid lesions i.e atheromas. They can lead to ischemic lesions of the heart, brain or extremities and can result in thrombosis and infarction of affected vessels, leading to death [4]. Lawrence T. Glickman et al evaluated the risk of endocarditis and other cardiovascular events on the basis of the severity of periodontal disease in dogs and found significant associations between the severity of periodontal disease and the subsequent risk of cardiovascular-related conditions [5]. Periodontal pathogens and their products have been reported to trigger the atherosclerotic process in animal and human studies [6, 7]. Raise in the levels of CRP have been commended to envisage potential development of CHD (Coronary heart disease) [8].Research suggested that periodontal disease, once established, outfits a biological burden of endotoxin and inflammatory cytokines like TNF-945; which succor to kick off and intensify atherogenesis and thromboembolic proceedings [9]. Investigators added to substantiate the link between sepsis and TNF by utilizing anti-TNF monoclonal antibodies to counteract the circulating TNF and thereby put off its adverse effects on cardiovascular system [10]. This goads us to guesstimate the role of periodontal therapy as voguish and empirical model to debate systemic inflammation in the above background.

The aim of this study is to investigate the alteration in serum inflammatory biomarker levels (CRP & TNF-α) in cardiovascular patients before and after periodontal therapy.

## Methods

A total number of 40 subjects were selected from Dept of periodontics, Kamineni institute of dental sciences and Apurv diagnostic and scan centre, Nalgonda, India. The sample size was calculated based on prior information from a pilot study conducted by our research group, Ethical committee approval was taken from the hospital (KIDS/Perio/08-11), the nature and intention of the study was explained to the patients and an informed consent was obtained and randomly allocated to one of the groups. A detailed case history was recorded in a specially prepared proforma which included information about the patient's overall medical status/general health, oral status and well-being.

The patients were divided into 2 groups

### Group A (Control group)

20 subjects (15 male and 5 female) within age group 45-70 years (mean age 55.80 ± 6.62) with probing depth of ≥ 5mm, where no treatment was given.

### Group B (Test group)

20 subjects (15 male and 5 female) within age group 45-70 years(mean age 56.13 ± 6.86) with probing depth of ≥ 5mm, where scaling and root planing (SRP) was performed. Inclusion criteria were subjects with Myocardial infraction (MI) aged between 45-70 years, who have not undergone any extractions or periodontal therapy in the last 6 months, with a minimum of 20 teeth in the oral cavity. Exclusion criteria were subjects with pregnancy, deleterious habits like smoking/alcohol consumption, subjects on any medication known to effect the serum levels such as antibiotics, Non-Steroidal Anti-Inflammatory Drugs (NSAIDs) in the past 6 months, Subjects having any other systemic diseases other than MI. After the selection of subjects a detailed case history was taken and the clinical parameters like probing pocket depth (PPD), Oral hygiene index simplified (OHI-S) were recorded. These parameters are assessed for subjects in 2 groups. At a baseline visit, a blind examiner collected a complete medical history, standard clinical periodontal parameters and blood samples. For each subject, the periodontal disease status was evaluated at 4 sites per tooth (Buccal, Distobuccal, Mesiobuccal and lingual) by an individual skilled periodontist using UNC-15 probe (Hu-Friedy′s, USA). Every subject was assessed twice in one visit, over a 30 min period, as mentioned above. The subsequent set of measurements was carried out blinded to the primary assessment. Measurements were made to the nearest millimeter and, where in doubt the lesser value was scored. Eighty five per cent of site measurements were frequent with an accuracy of ± 1 mm and seventy percent of sites were recorded accurately to the similar value in both the assessments. All subjects in group B received a thorough session of subgingival periodontal therapy consisting of mechanical instrumentation of the entire diseased dentition under local anaesthesia once a week for 3 weeks. SRP were performed by Gracey Curettes along with ultra-sonic instruments. SRP was not accompanied by any medications such as antibiotics, non-steroidal anti-inflammatory drugs or mouthwash. For the assessment of CRP and TNF-α blood samples were collected from subjects at the time of clinical examination and after 8 weeks for reassessment in group A and group B subjects.

### Blood sample collection and storage

About 10 ml of blood sample was collected from each of the subjects from the brachial vein, by aseptic technique using a 10cc syringe and transferred to an appropriately labeled tube, centrifuged for 10min at 3,000 RPM separating the cells from the serum and the smear layer was removed carefully. The serum thus obtained was stored at -80oC for the analyses at a later date. These specimens were stored so that the evaluation of samples which would be taken on the completion of the study could be done concurrently to minimize wear and tear of the wells in the kit.

### CRP and TNF alpha quantification

Serum C - reactive protein levels were assessed by means of High Sensitivity C - reactive protein (hs-CRP) Enzyme Immunoassay *(Thyrocare and Scopelabs - Nephlometric method)*. Serum TNF-α assessed by means of a commercially available ELISA kit from ISO certified ^9001 / 13485^ IMMUNOTECH A Beckham Coulter Company^®^ France, Ref Im 1121- Im 11121. These assays have a lower limit of detection for TNF-α of approximately 20 pg/dl, for CRP 0.3 µg/dl.

### Calculations

The sample results are calculated by interpolation from a calibrator curve that is performed in the same assay as that of the sample. The curve is drawn, plotting on the horizontal axis the TNFα concentration of the calibrator and on the vertical axis the corresponding absorbance. The absorbance for each sample is on the vertical axis and read off the corresponding TNFα concentration on the horizontal axis.

### Statistical methods

The data was analysed using SPSS 17 (Statistical package for the social sciences), Microsoft word and Excel have been used to generate Figures, tables etc. Mean and standard deviation are calculated for all the parameters (CRP, TNF-α, OHI-S, PPD) of both the groups at baseline and after 8 weeks. Continuous data were expressed as mean ± SD. Mean values of each parameter are compared between the groups using paired sample t test. A P-value of < 0.05 was considered for statistical significance, P-value < 0.001 was considered statistically highly significant. Relationships between the parameters were assessed by Pearson's correlation coefficient.

## Results

In total 39 people completed the study(1 patient in Group A lost follow up). All the patients who participated in the study were MI patients and were adjusted for factors known (other than MI) to elevate CRP & TNF-α level. There were no significant differences between groups at baseline in terms of age, number of teeth, reported oral hygiene.

### Age, sex & BMI distribution among the groups

**Figure 1:**
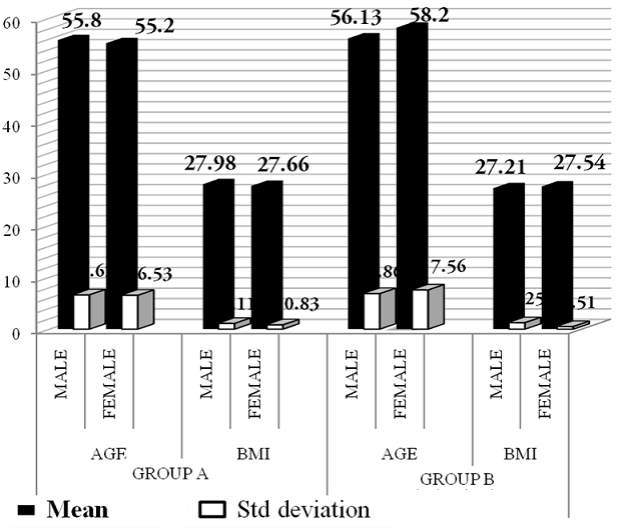
Age, sex & BMI distribution among the groups

The mean age of the subjects in Group A is 55.80 ± 6.62 among 15 males & 55.20 ± 6.53 among 5 females. In Group B 56.13 ± 6.86 among 15 males & 58.20 ± 7.56 among 5 males. The mean BMI distribution in Group A is 27.98 ± 1.11 among 15 males and 27.66 ± 0.83 among 5 females. In Group B 27.21 ± 1.25 among 15 males and 27.54 ± 0.51 among 5 females (Table 1 and Figure 1).

**Table 1:** Demographic data at baseline

	Group A (n = 20)		Group B (n = 20)		Group A vs Group B
	M (n = 15)	F (n = 5)	M (n = 15)	F (n = 5)	P value
AGE¶	55.80 ± 6.62	55.80 ± 6.62	56.13 ± 6.86	56.13 ± 6.86	0.762#
BMI¶	27.98 ± 1.11	27.98 ± 1.11	27.21 ± 1.25	27.21 ± 1.25	0.559#

¶ Equal variances assumed

# Statistically not significant (P > 0.05)

### Periodontal status

OHI-S: With a thorough basic therapy followed by regular maintenance, the plaque control in all the patients was satisfactory. At baseline the OHI-S index in the Groups A and B was 4.45 ± 0.65, 4.74 ± 0.7 respectively. After 2 months the OHI - S index in the Group A was 4.85± 0.62 and Group B was 1.5±0.4.

**Figure 2:**
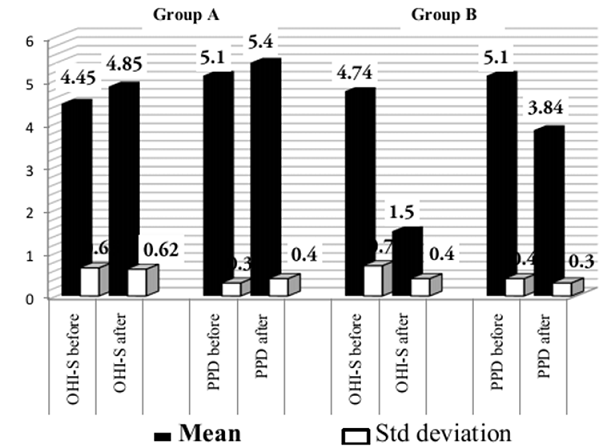
Mean periodontal scores of two groups

There was improvement in Group B and was found to be statistically highly significant (P < 0.001), whereas in Group A there was no improvement in OHI-S index. Comparing Group A and Group B, the reduction OHI-S index was found to be better in Group B as SRP was performed (Table 2 and Figure 2).

**Table 2:** Preoperative and postoperative clinical and chemical data for Group A and B

	Group A (n = 19)			Group B (n = 20)		
	Preoperative	Postoperative	Preoperative Vs Postoperative *(Sig 2 tailed)*	Preoperative	Postoperative	Preoperative Vs Postoperative *(Sig 2 tailed)*
OHI-S	4.45± 0.65	4.85 ± 0.62	.001*¶	4.74 ± 0.7	1.5 ± 0.4	.001**
PPD	5.10 ± 0.3	5.4 ± 0.4	.001*¶	5.10 ± 0.4	3.84 ± 0.26	.001**
CRP (µg/dl)	0.47±0.11	0.45 ± 0.14	.141#	0.45 ± 0.12	0.29 ± 0.12	.001**
TNF-α (pg/ml)	22.85± 1.29	22.68 ± 1.23	.056#	22.14 ± 1.46	20.20 ± 1.61	.001**

** Statistically highly significant (P < 0.05)

*¶ Statistically highly significant where t is negative

# Statistically not significant (P > 0.05)

### Probing pocket depth

**Figure 3:**
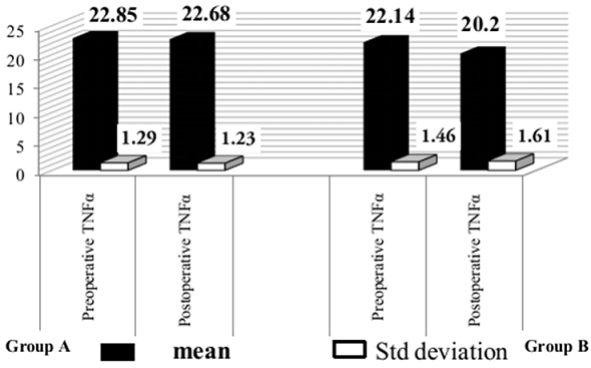
Mean TNF-α level of the groups

Periodontal pockets vary in their location and depth; hence changes in the mean probing depths for the entire mouth provide realistic information. Accordingly, the mean probing depths for the entire mouth at the beginning of the study for Groups A and B were 5.10±0.30 mm and 5.10±0.4mm respectively. After 8 weeks, the mean PPD in Group A and B are 5.4±0.4mm and 3.84±0.26 mm respectively. Mean PPD was found to be decreased in Group B and was highly statistically significant (P < 001). In Group A there was no improvement in the PPD as there was no treatment performed. (Table 2 and Figure 3).

**Table 3:** Pearson's correlation between Changes in CRP and TNF -α with Changes in Periodontal parameters

Pearson correlation coefficient (P value)	GROUP A	GROUP B	Overall
Change in CRP and Change in OHI-S	0.096 (0.697)	0.430 (0.058)	0.551 (0.000)
Change in CRP and Change in PPD	-0.390 (0.099)	-0.285 (0.224)	0.366 (0.022)
Change in TNF-α and Change in OHI-S	-0.074(0.763)	0.135 (0.570)	0.641 (0.000)
Change in TNF-α and Change in PPD	-0.040(0.871)	0.057 (0.813)	0.613 (0.000)

### C reactive protein

**Figure 4:**
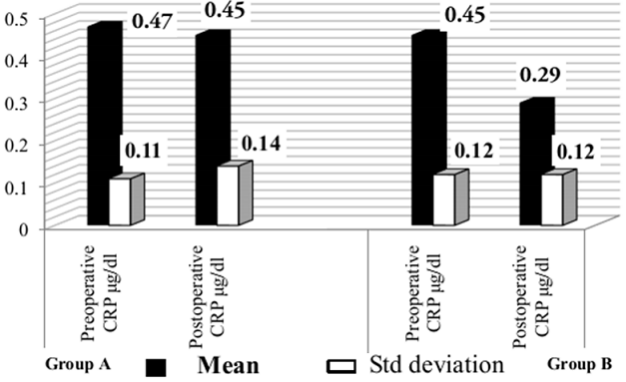
Mean CRP level of the groups

The baseline CRP concentrations in the Groups A and B were 0.47±0.11µg/dl, 0.45±0.12µg/dl respectively. After 8 weeks, the CRP level in Group A and B reduced to 0.45±0.14µg/dl and 0.29±0.12µg/dl respectively. This was statistically highly significant (P < 0.001) in Group B. Whereas in Group A there was minimal decrease in CRP level which is not statistically significant (P > 0.05). (Table 2 and Figure 4).

### TNF-α

**Figure 5:**
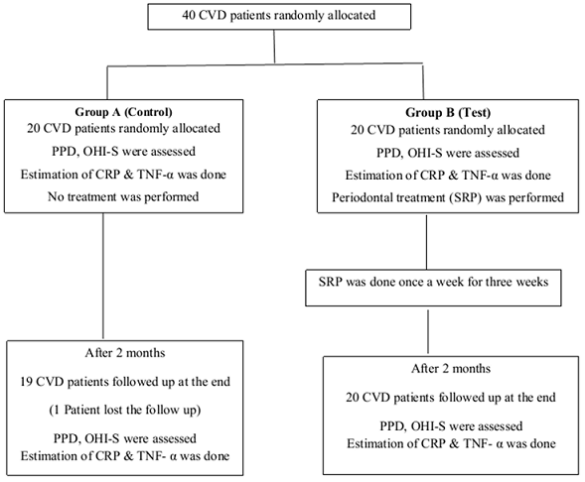
Experimental design of 40 subjects

The baseline TNF-α concentrations in the Groups A and B were 22.85±1.29pg/dl, 22.14±1.46pg/dl respectively. After 8 weeks, the TNF-α level in Group A and B reduced to 22.68±1.23pg/dl and 20.20±1.61pg/dl respectively. This was statistically highly significant (P < 0.001) in Group B. Whereas in Group A there was minimal decrease in TNF-α which is not statistically significant (P > 0.05). (Table 2 and Figure 5).

## Discussion

The present study reports the outcome by examining the effects of non-surgical periodontal treatment on systemic markers of inflammation and the results are interesting and important to those with closely related research as it established that effective management of periodontal infection decreased serum inflammatory markers (CRP, TNF-α) in a comparatively small population (N = 39) with chronic generalized periodontitis.

Why the interest in proinflammatory cytokines TNF-α and CRP; why are we thinking about lowering inflammation to lower vascular risk? In one meta-analysis the findings resulted in a conclusion that periodontitis and poor oral health overall indeed contribute to the pathogenesis of cardiovascular disease [11]. CRP,TNF-α and additional acute-phase molecules are typically present at relatively low levels in plasma, but may be raised significantly with tissue injury or various bacterial infections together with periodontitis.In the present study, clinical parameters such as OHI-S index and PPD showed a positive correlation with CRP & TNF-α level in both the groups with Chronic Periodontitis.

These results were also in agreement to the findings of Noack B., et al [3] who stated that the level of raise in CRP levels in periodontitis patients relies on the severity of the disease after adjusting for age, BMI, smoking and it was also stated that raise in CRP levels were seen coupled with infections of subgingival organisms and is frequently connected with periodontal disease [3].In studies of patients following an MI, those who have high TNF-α levels have the highest rate of recurrent cardiovascular events [12]. CRP is one of the most sensitive acute - phase reactants. It increases rapidly in response to many disease conditions.

The present study was envisaged to determine the influence of periodontal treatment on the serum CRP & TNF-α level in cardiovascular patients with periodontitis. The outcome of the present study demonstrated a raise in serum CRP & TNF-α level concomitantly with the severity of the disease and a appropriately performed periodontal therapy results in the improvement of the periodontal parameters irrespective of the condition of the disease. Clinical parameters such as OHI-S index and PPD showed a positive correlation with CRP & TNF-α level in both the groups with chronic periodontitis in this study. This is similar to the results of earlier studies which revealed that increased bleeding on probing, probing depth and attachment loss to be significantly associated with elevated CRP & TNF-α concentrations [2, 3, 13, 14].The present study showed that CRP & TNF-α level in Group B who received periodontal treatment decreased significantly [14–19].

Other studies which have failed to confirm decrease in CRP & TNF-alpha with periodontal treatment should however, be mentioned [2, 20]. The study by Ide et al did not account for the effects of obesity, hypertension and cholesterol and it is possible that these confounders may have influenced their findings. Also after the periodontal treatment some residual disease sites remained and this may have had some bearing on the results obtained in their study [2].

While Ebersole et al. observed a relationship between CRP levels and the presence or absence, or severity of, adult periodontitis, it is not evident whether their study design controlled for the effects of smoking, a confounder for both periodontitis and serum markers concentration [21]. Ebersole et al. also not successful to observe a reduction in circulating CRP following nonsurgical periodontal treatment, although there was no indication of the efficacy of this treatment. In the study by Yamazaki et al, although the CRP level in periodontitis patients tended to decrease with improvement of the periodontal condition following treatment and approached that of the control subjects, this decline was not statistically significant [22].The results of the present study reinforce the observations of the previous studies indicating that periodontal disease is associated with elevation in serum CRP & TNF-α level. Therefore elevation of CRP & TNF-α such as that seen in periodontal disease may supplement systemic vascular inflammation, atheroma formation and add to the pre existing risk for cardiovascular sequelae. If periodontitis can lead to the elevation of CRP & TNF-α levels, then theoretically, periodontal therapy should help in reducing the systemic burden of inflammation. However, to know whether such benefit can really be translated in the long term, can only be assessed by well controlled longitudinal clinical trials. Therefore, further studies should focus on the relationship between periodontitis, elevated CRP & TNF-α levels and the effect of periodontal therapy on serum inflammatory markers concentration.

## Conclusion

Within the limitations of this study, it may be concluded that the clinically successful non-surgical periodontal therapy tend to reduce concentration of circulating pro inflammatory cytokines (CRP, TNF-α), which could be important for cardiovascular disease. This beneficial effect of periodontal therapy appears to be available irrespective of the degree of periodontal destruction seen at the baseline. Properly powered longitudinal case-control and intervention trials are needed to identify how periodontitis and periodontal interventions may have an impact on cardiovascular diseases.
